# Population attributable fraction of risk factors for anemia among children aged 6–59 months: A multilevel complex data analysis using modified poisson regression model in Sub-Saharan Africa

**DOI:** 10.1371/journal.pone.0324835

**Published:** 2025-06-25

**Authors:** Meklit Melaku Bezie, Beminate Lemma Seifu, Hiwot Altaye Asebe, Angwach Abrham Asnake, Yohannes Mekuria Negussie, Zufan Alamrie Asmare, Mamaru Melkam, Bezawit Melak Fente

**Affiliations:** 1 Department of Public Health Officer, Institute of Public Health, College of Medicine and Health Sciences, University of Gondar, Gondar, Ethiopia; 2 Department of Public Health, College of Medicine and Health Sciences, Samara University, Samara, Ethiopia; 3 Department of Epidemiology and Biostatistics, School of Public Health, College of Medicine and Health Sciences, Wolaita Sodo University, Wolaita Sodo, Ethiopia; 4 Department of Medicine, Adama General Hospital and Medical College, Adama, Ethiopia; 5 Department of Ophthalmology, School of Medicine and Health Science, Debre Tabor University, Debre Tabor, Ethiopia; 6 Department of Psychiatry, University of Gondar College of Medicine and Health Science, Gondar, Ethiopia; 7 Department of General Midwifery, School of Midwifery, College of Medicine & Health, Sciences, University of Gondar, Gondar, Ethiopia; PLOS: Public Library of Science, ETHIOPIA

## Abstract

**Background:**

Anemia among children under five remains a significant public health concern in sub-Saharan Africa (SSA). While numerous studies have examined its associated factors, limited evidence exists on the public health impact of modifiable risk factors. This study assessed the population-attributable fraction (PAF) of risk factors for anemia among children aged 6–59 months across 27 SSA countries.

**Methods:**

A secondary data analysis was conducted using Demographic and Health Survey (DHS) data from 27 SSA countries, including a weighted sample of 124,285 children. Data management and analysis were done using STATA-14 software. The presence of clustering was assessed using the Intra-cluster Correlation Coefficient (ICC), Median Odds Ratio (MOR), and Likelihood Ratio Test (LRT). A multilevel modified Poisson regression model was employed to estimate adjusted Prevalence Odds Ratios (aPOR) and the corresponding PAF with 95% Confidence Intervals (CI), and the Population Attributable Fraction (PAF) with the 95% CI was estimated using the aPOR and the prevalence of exposure.

**Results:**

The overall prevalence of anemia among children in SSA was 61.99% (95% CI: 61.73, 62.27). Significant risk factors included maternal anemia (PAF = 7.37%), low maternal education (PAF = 5.86% for no formal education), poor household wealth status (PAF = 3.52% for the poorest), lack of media exposure (PAF = 0.76%), child undernutrition (stunting PAF = 1.31%, underweight PAF = 0.84%), diarrheal (PAF = 1.19%) and febrile illness (PAF = 2.26%), and unimproved toilet facilities (PAF = 1.02%).

**Conclusion:**

Maternal anemia, low maternal educational status, poverty, poor sanitation, inadequate media access, unimproved toilet facilities, and childhood illness significantly contribute to anemia in SSA. Targeted interventions to improve maternal and child health, enhance education, and ensure better nutrition and sanitation could help reduce childhood anemia in the region.

## Background

Anemia is a critical public health issue affecting children and is widely prevalent, especially among those under the age of five, with a high burden in developing countries like Sub-Saharan Africa [1]. The World Health Organization (WHO) defines anemia in under-five children as a hemoglobin level less than 11 mg/dL [2]. Over 269 million children were anemic annually, accounting for 43% of all children worldwide, half of whom lived in low- and middle-income countries [3]. Nearly two-thirds (64.1%) of children in sub-Saharan Africa (SSA) were anemic [4].

In SSA, nutritional deficiency anemia is the most common form of anemia, especially in under-five children [5,6]. It is linked to the tremendous burden of undernutrition and infectious diseases in SSA [7]. Poor dietary intake, infectious diseases such as malaria and schistosomiasis, mothers’ poor nutritional status, and insufficient maternal health services all contribute to the high prevalence of anemia. The high prevalence of anemia is exacerbated by poor dietary intake, infectious diseases like malaria and schistosomiasis, the poor nutritional status of mothers, and inadequate maternal health services [8–10]. Anemia has a significant impact on the cognitive growth and development [11,12]. It negatively impacts children’s growth and motor development, which leads to delayed speech [13,14]. It may result in immediate as well as long-term consequences [15].

Iron deficiency anemia is one of the top ten morbidity cases in the world, limiting a child’s ability to complete their education by impairing concentration, memory, and learning capacity [5]. Furthermore, anemia causes emotional instability among young children, as well as problematic and reckless behavior in school [16]. Besides, a child with anemia is more vulnerable to infections [17,18]. The long-term consequences can impact social interactions in adulthood, and lead to economic challenges [19,20]. In later life, anemic mothers may experience preterm births and low birth weight deliveries, accounting for 20 million deaths of children every year [21–23].

Despite international efforts to combat and reduce its prevalence, anemia remains a burden, particularly in Sub-Saharan Africa and South Asia [24,25]. Sub-Saharan Africa has implemented health measures such as food fortification, deworming, and iron/folate supplementation to reduce the incidence of anemia in children [26]. These initiatives are consistent with the Sustainable Development Goals (SDGs), which seek to improve child health and ultimately save lives [27]. In previous studies conducted on anemia among under-five children numerous child-related, maternal- and health service-related factors were found strong predictors of anemia among under-five children [28,29]. Factors such as sex of child [30], use of insecticide-treated bed nets [31], birth order [32], child age [33], and micronutrient deficiencies (folic acid, zinc, and vitamin B12) [34], maternal education, parental education [35], employment status, residence [36], maternal age [37], media exposure [38], economic status [4,39], and maternal anemia [40] were significantly associated with anemia among under-five children.

Numerous studies have been published on factors associated with anemia among children aged 6–59 months in sub-Saharan Africa [1,4,41,42]. However, none of these studies have assessed the public health significance of risk factors for anemia among children aged 6–59 months in sub-Saharan Africa (SSA) using the Population Attributable Fraction (PAF). The PAF is crucial for identifying and prioritizing modifiable risk factors for anemia, which can guide the implementation of effective intervention programs. By pinpointing these key risk factors, targeted strategies can be developed to reduce the burden of anemia and lower anemia-related mortality among children in this age group, ultimately contributing to the achievement of the Sustainable Development Goals (SDGs). Therefore, this study aimed to estimate the population-attributable fraction of risk factors for anemia among children aged 6–59 months in SSA.

## Methods and materials

### Data source

The Demographic and Health Survey (DHS) data of 27 sub-Saharan African countries conducted from 2014 to 2022 were used. Countries included were Angola, Burkina Faso, Benin, Burundi, Democratic Republic of Congo, Cote d’Ivoire, Cameroon, Ethiopia, Gabon, Ghana, Gambia, Guinea, Liberia, Lesotho, Madagascar, Mali, Mauritania, Malawi, Mozambique, Nigeria, Rwanda, Serra Leone, Togo, Tanzania, South Africa, Zambia and Zimbabwe. The Kids Record (KR) dataset of these countries was used to investigate the population-attributable fraction of risk factors of anemia among under-five children. A multistage stratified sampling technique was employed to recruit study participants for the survey using the Enumeration Area (EA) served as the primary sampling unit, while households were the secondary sampling unit. In the first stage, the administrative state was classified into urban and rural areas within each region, and enumeration areas (EAs) were then selected proportionally to the population size of each stratum. A complete household listing was conducted in each selected cluster. In the second stage, is the systematic sampling of households listed in each cluster. To assess the anemia status of children aged 6–59 months in the selected household, the HemoCue rapid testing methodology was used. A drop of capillary blood was drawn from a child’s fingertip or heel and placed in a micro cuvette, which was then analyzed with a photometer to determine the hemoglobin concentration. The detailed methodology is available in the Guide to DHS statistics manual [43]. A total weighted sample of 124,285 children aged 6–59 months was used.

### Outcome variable

Anemia was the outcome variable. It was defined as anemic (hemoglobin level was < 11.0g/dl) and non-anemic (hemoglobin ≥11.0 g/dl) [44]. It was determined using the hemoglobin concentration in the blood, which was adjusted for altitude.

### Modifiable risk factors

Given the study’s objective of estimating the population-attributable fraction of risk factors for anemia, we were particularly interested in modifiable risk factors. The selection of these factors was based on a comprehensive review of relevant literature, existing theoretical frameworks, and the availability of data in the survey. Factors were considered modifiable if previous studies and public health guidelines suggested that targeted interventions could alter their effects on anemia outcomes. Risk factors such as maternal education, maternal occupation, media exposure, household wealth status, marital status, maternal anemia, preceding birth interval, history of diarrhea, history of fever, child nutritional status (stunting, wasting, and underweight), taking drugs for the intestinal parasite in the last 6 months, exclusive breastfeeding, antenatal care visit, birth size, place of delivery, type of toilet facility and water source were considered. Stunting is defined as the children with Height-for-Age Z-score (HAZ) <−2SD, wasting is defined as the children with Weight-for-Height Z-score (WHZ) <−2SD, and underweight is defined as the children with Weight-for-Age Z-score (WAZ) <−2SD [45].

### Confounding variables

Covariates included were sex of child, number of gestations for the index birth (grouped as single and twin), parity (grouped as < 5 and> = 5 births), number of under-5 children in the household (grouped as <= 2 and > 2 under-five children), household size (grouped as 2–4, 5–7, and> = 8), place of residence, and maternal age (grouped as 15–24 years, 25–34 years and> = 35 years).

### Data management and analysis

All the analysis was based on the weighted data adjusted for design and non-response using sampling weight, primary sampling unit, and strata. We used the STATA 14 software for data management and analysis. Owing to the hierarchical nature of DHS and a typical statistical method the multilevel modified poisson model was used. In a cross-sectional study, reporting the Odds Ratio (OR) could overestimate the relationship between the independent and dependent variables. Therefore, the Prevalence Odds Ratio (POR) is the best measure of association for the current study. To obtain the POR, a multilevel modified poisson model was fitted. The Median Odds Ratio (MOR), Likelihood Ratio (LR) test, and Intra-class Correlation Coefficient (ICC) were computed to measure the variation between clusters. The ICC quantifies the degree of heterogeneity between clusters (the proportion of the total observed individual variation in anemia among children 6–59 months attributable to cluster variations) [46].


$$ICC=\sigma^{2}/\left(\sigma^{2}+\frac{\pi^{2}}{3}\right)$$

π^2^/3 is the individual-level variance approximated to 3.29, and $\sigma^{2}$ represents the community-level variance. MOR is quantifying the variation or heterogeneity in outcomes between clusters. It is defined as the median value of the odds ratio between the cluster at higher risk of anemia and the cluster at lower risk when randomly picking out two clusters (EAs) [47].


$$\begin{array}{c} \text{MOR}=\exp\left(\sqrt{2*\partial 2*0.6745}\right)\sim \text{MOR}=\text{exp}(0.95*\partial ). \end{array}$$

A multivariable multilevel modified poisson model was employed to estimate the adjusted Prevalence Odds Ratio (aPOR).

The Population Attributable Fraction (PAF) was calculated for the significant modifiable risk factors to estimate the contribution of each risk factor to the total risk for anemia using the **Punaf** Stata commands (S1 File). The PAF describes the proportion of anemia cases among children aged 6–59 months that could be prevented if the modifiable risk factors were eliminated in the population. PAF determines the proportion of anemia among children aged 6–59 months in SSA that could be prevented by eliminating the modifiable risk factor among the population [48]. It was calculated using the Levin’s formula;


$$PAF=\frac{Pr*(aPOR-1)}{1+(Pr*(aPOR-1)}$$

Where Pr represents the risk factor’s prevalence in the population and aPOR represents the exposure category’s adjusted prevalence odds ratio. This formula provides unbiased estimates of PAF in the presence of confounding variables [49]. Because the risk factors frequently occur together within individuals, adding the PAFs of each risk factor would result in an overestimation of their combined PAFs. We used the formula to calculate a joint PAF across all risk factors.


$$PAF(combined)=1-\prod_{r=1}^{R}\left(1-PAFr\right)$$

Where r denotes each exposure variable. The assumption that exposures are independent and uncorrelated has been reduced by the use of prevalence odds ratios that have been adjusted for potential confounders. This is consistent with the classification of the counterfactual distribution of modifiable risk factors proposed by Murray and Lopez [50].

### Ethics approval

As the study was a secondary data analysis of publicly accessible survey data from the MEASURE DHS program, this study did not require ethical approval and participant consent. We have granted permission from http:/www.dhsprogram.com to download and use the data for this study. In the datasets, there are no names of persons or household addresses recorded.

## Results

### Baseline characteristics and prevalence of anemia

**Fig 1:**
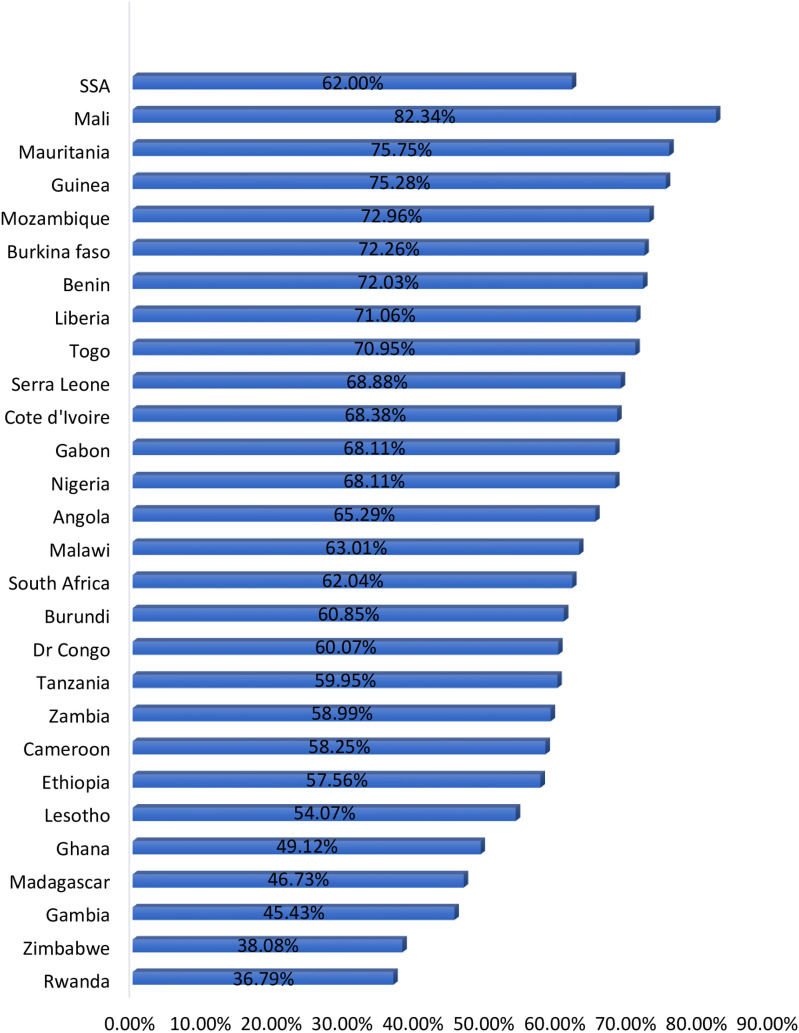
The prevalence of anemia among children aged 6–59 months in sub-Saharan African countries.

A total of 124,285 children aged 6–59 months in SSA were included. More than one-third (36.63%) and 39,918 (32.12%) of the mothers had no formal and primary education, respectively. About 31,634 (25.45%) and 60,893 (48.99%) of the children were born to mothers aged 15–24 and 25–34 years, respectively. A higher prevalence of anemia among mothers who had no formal education (68.82%), primary education (60.54%), mothers aged 15–24 years (66.88%), and 25–34 years (61.15%). Regarding household wealth status, more than two-thirds (68.57%) of children belonged to the poorest household and nearly two-thirds (64.78%) of children who belonged to the poorer household had anemia (Table 1). The prevalence of anemia among children aged 6–59 months in SSA was 61.99% (95% CI: 61.73, 62.27). It ranged from 36.79% in Rwanda to 82.34% in Mali (Fig 1).

**Table 1:** Baseline characteristics of the study population and the prevalence of anemia among children aged 6–59 months in SSA.

	Study population		Anemia		
**Freq.(n)**	**%**	**Freq. (n)**	**%**	**95% CI**
**Maternal education status**					
No	45,525	36.63	31,331	68.82	[68.39, 69.24]
Primary	39,918	32.12	24,165	60.54	[60.06, 61.01]
Secondary	34,247	27.55	19,406	56.67	[54.14, 57.19]
Higher	4,595	3.70	2,152	46.84	[45.40, 48.28]
**Maternal age**					
15 −24	31,634	25.45	21,156	66.88	[66.36, 67.40]
25–34	60,893	48.99	37,234	61.15	[60.76, 61.53]
≥ 35	31,759	25.55	18,665	58.77	[58.23, 59.31]
**Household wealth status**					
Poorest	28,378	22.83	19,460	68.57	[68.03, 69.11]
Poorer	26,856	21.61	17,396	64.78	[64.20, 65.35]
Middle	25,203	20.28	15,666	62.16	[61.56, 62.76]
Richer	23,852	19.19	14,176	59.44	[58.82, 60.06]
Richest	19,996	16.09	10,354	51.78	[51.09, 52.47]
**Media exposure**					
No	47,466	38.19	31,163	65.65	[65.22,66.08]
Yes	76,820	61.81	45,891	59.74	[59.39,66.08]
**Residence**					
Rural	42,861	34.49	25,031	58.40	[57.93,58.87]
Urban	81,424	65,51	52,023	63.89	[63.56,64.22]
**Child age (in months)**					
6–11	14,953	12.03	11,325	75.73	[75.04,76.41]
12–23	29,358	23.62	21,176	72.13	[71.62,72.64]
24–59	79,974	64.35	44,553	55.71	[55.37,56.05]
**Maternal anemia**					
No	67,135	54.02	36,726	54.71	[54.33, 55.08]
Yes	49,460	39.80	35,256	71.28	[70.88, 71.68]
Not stated	7,690	6.19	5,072	65.96	[64.89, 67.01]
**Stunting**					
No	83,032	66.81	49,336	59.42	[59.08,59,75]
Yes	41,253	33.19	27,718	67.19	[66.74,67.64]
**Wasting**					
No	117,089	94.21	72,096	61.57	[61.30,61.85]
Yes	7,197	5.79	4,958	68.89	[67.81,69,94]
**Underweight**					
No	103,320	83.13	62,399	60,39	[60.10,60,69]
Yes	20,965	16.87	14,655	69,90	[69,28,70.52]
**Sex of child**					
Male	62,845	50.57	39,866	63.43	[63.06,63.81]
Female	61,440	49.43	37,187	60.53	[60.14,60.91]
**Number of gestations for the index birth**					
Single	120,404	96.88	74,525	61.90	[61.62,62.17]
Twin	3,882	3.12	2,529	65.15	[63.64,66.64]
**Birth spacing**					
≤ 24	20,370	16.39	13,133	64.47	[63.81,65.13]
> 24	76,775	61.77	47,703	62.13	[61.79,62.48]
First birth	27,140	21.84	16,219	59.76	[59.18,60.34]
**History of diarrhea within 2 weeks**					
No	105,601	84.97	64,196	60.79	[60.49, 61.08]
Yes	18,684	15.03	12,859	68.82	[68.15, 69.48]
**History of fever within 2 weeks**					
No	98,175	78.99	58,749	59.84	[59.53, 60.15]
Yes	26,110	21.01	18,306	70.11	[69.55, 70.66]
**Marital status**					
Married	108,461	87.27	67,443	62,18	[61.89,62.47]
Not married	15,825	12.73	9612	60.74	[59.98,61.50]
**Parity**					
< 5 births	83,135	66.89	50,903	61.23	[60.90,61.56]
≥ 5 births	41,150	33.11	26,151	63.55	[63.08,64.01]
**Birth size**					
Larger	37,691	30.33	23,403	62.09	[61.60, 62.58]
Average	57,145	45.98	35,707	62.48	[62.09, 62.88]
Smaller	17,113	13.77	10,984	64.18	[63.46, 64.89]
Not stated	12,336	9.93	6,961	56.43	[55.55, 57.30]

### Multivariable analysis of risk factors of anemia

The multivariable multilevel modified Poisson analysis result shows that variables such as maternal educational status, wealth status, media exposure, maternal anemia, diarrhea and fever within two weeks, and unimproved toilet facilities had a statistically significant association with anemia. Children born to mothers who had no formal, primary and secondary education 1.17 (aPOR = 1.17, 95% CI: 1.11, 1.23), 1.12 (aPOR = 1.12, 95% CI: 1.07, 1.18) and 1.10 (aPOR = 1.10, 95% CI: 1.05, 1.16) times higher prevalence odds of anemia than those born to mothers who had higher education, respectively. The odds of anemia among children belonged to poorest, poorer, middle and richer 1.16 (aPOR = 1.16, 95% CI: 1.12, 1.20), 1.12 (aPOR = 1.12, 95% CI: 1.09, 1.16), 1.10 (aPOR = 1.10, 95% CI: 1.07, 1.14) and 1.08 (aPOR = 1.08, 95% CI: 1.05, 1.11) times higher compared to those belonged to richest household, respectively. Children born to mothers who had no media exposure had 1.02 times (aPOR = 1.02, 95% CI: 1.00, 1.03) higher prevalence odds of anemia compared to those born to mothers who had media exposure. Maternal anemia increased the prevalence odds of anemia among children by 1.20 (aPOR = 1.20, 95% CI: 1.18, 1.22). Stunting and underweight increased the prevalence odds of anemia among children by 1.04 (aPOR = 1.04, 95% CI: 1.02, 1.08) and 1.05 (aPOR = 1.05, 95% CI: 1.02, 1.07) times higher, respectively.

The prevalence odds of anemia among children who had diarrhea and fever within two weeks were 1.08 (aPOR = 1.06, 1.10 CI:1.06,1.10) and 1.11 (aPOR = 1.11, 95% CI: 1.09, 1.13) times higher compared to those who didn’t have diarrhea and fever within two weeks, respectively. Unimproved toilet facilities increased the prevalence odds of anemia among children by 1.04 (aPOR = 1.04, 95% CI: 1.02, 1.06) compared to those with improved toilet facilities (Table 2).

**Table 2:** Adjusted POR from the multilevel modified poisson regression and population attributable fractions for significant risk factors associated with anemia among children aged 6–59 months in SSA.

Variables	Proportion of exposure	CI	aPOR	CI	PAF %	CI
**Maternal education status**						
No	36.63	[36.36, 36.89]	1.17	[1.11, 1.23]	5.86	[3.85, 7.82]
Primary	32.12	[31.86, 32.38]	1.12	[1.07, 1.18]	3.71	[2.18, 5.51]
Secondary	27.55	[27.31, 27.80]	1.10	[1.05, 1.16]	2.68	[1.35, 4.26]
Higher	3.67	[3.60, 3.80]	1		1	
**Household wealth status**						
Poorest	22.83	[22.60, 23.07]	1.16	[1.12, 1.20]	3.52	[2.64, 4.41]
Poorer	21.61	[21.38, 21.84]	1.12	[1.09, 1.16]	2.53	[1.89, 3.38]
Middle	20.28	[20.06, 20.50]	1.10	[1.07, 1.14]	1.99	[1.38, 2.79]
Richer	19.19	[18.97, 19.41]	1.08	[1.05, 1.11]	1.51	[0.94, 2.09]
Richest	16.09	[18.89, 16.29]	1		1	
**Media exposure**						
No	38.19	[37.92, 38.46]	1.02	[1.00, 1.03]	0.76	[0.01, 1.14]
Yes	61.81	[61.54, 62.08]	1		1	
**Maternal anemia**						
No	54.02	[53.74, 54.29]	1		1	
Yes	39.80	[39.52, 40.07]	1.20	[1.18, 1.22]	7.37	[6.64, 8.10]
**Stunting**						
No	66.81	[66.55, 67.07	1		1	
Yes	33.19	[32.93, 33.45]	1.04	[1.02, 1.08]	1.31	[0.6, 2.61]
**Wasting**						
No	94.21	[94.08, 94.34]	1		1	
Yes	5.79	[5.66, 5.92]	1.02	[0.99, 1.05]	0.12	[-0.06, 0.30]
**Underweight**						
No	83.13	[82.92, 83.34]	1		1	
Yes	16.87	[16.66, 17.08]	1.05	[1.02, 1.07]	0.84	[0.33, 1.18]
**Birth spacing**						
> 24	61.77	[61.50, 62.04]	1		1	
≥24	16.39	[16.18, 16.6]	1.00	[0.98, 1.02]	0.01	[-0.16, 0.33]
First birth	21.84	[21.61, 22.07]	0.92	[0.90, 0.94]	−1.78	[-2.21, -1.34]
**History of diarrhea within 2 weeks**						
No	84.97	[84.77, 85.16]	1		1	
Yes	15.03	[14.84, 15.23]	1.08	[1.06, 1.10]	1.19	[0.88, 1.50]
**History of fever within 2 weeks**						
No	78.99	[78.76, 79.21]	1		1	
Yes	21.01	[20.78, 21.24]	1.11	[1.09, 1.13]	2.26	[1.84, 2.69]
**Type of toilet facility**						
Not improved	25.78	[25.54, 26.02]	1.04	[1.02, 1.06]	1	
Improved	74.22	[73.98, 74.46]	1		1.02	[0.51, 1.54]
**Type of water source**						
Not improved	55.35	[55.08, 55.63]	1		1	
Improved	44.65	[44.37, 44.92]	0.99	[0.98, 1.01]	−0.45	[-0.90, 0.45]

* *Abbreviation: aPOR = adjusted Prevalence Ratio, CI = Confidence Interval, PAF = Population Attributable Fraction*

### The goodness of fit of models in multilevel modified Poisson regression

The appropriateness, adequacy, and usefulness of the models were tested using Log-likelihood Ratio (LLR), deviance, and Likelihood Ratio Test (LRT). The ICC value in the null model was 10.41%, which showed that about 10.41% of the total variation in anemia among children aged 6–59 months in SSA was attributable to between cluster variation. Similarly, the MOR of the null model was 1.80, which means if we randomly move a child from a low-risk cluster to a high-risk cluster, the odds of having anemia would be increased by 1.80. Moreover, the LRT was statistically significant (p < 0.05), showing that the multilevel modified Poisson regression model fitted the data better than the standard regression model. We reported the results of the best-fitted model, a model with the lowest deviance value.

### Population attributable fractions for modifiable risk factors of anemia

Maternal educational status, household wealth status, media exposure, stunting, underweight, diarrhea in two weeks, fever in two weeks, and type of toilet facility significantly contributed to anemia among children aged 6–59 months. About 5.86% (PAF = 5.86%, 95% CI: 3.85, 7.82), 3.71% (PAF = 3.71%, 95% CI: 2.18, 5.51), and 2.68% (PAF = 2.68%, 95% CI: 1.35, 4.26) of anemia in children were attributable to mothers having no formal, primary and secondary education, respectively. Around 3.52% (PAF = 3.52%, 95% CI: 2.64, 4.41), 2.53% (PAF = 2.53%, 95% CI: 1.89, 3.38), and 1.99% (PAF = 1.99%, 95% CI: 1.38, 2.79) of anemia in children were attributable to being in the poorest, poorer and middle household wealth, respectively. About 0.76% (PAF = 0.76%, 95% CI: 0.01, 1.14) and 7.37% (PAF = 7.37%, 95% CI: 6.64, 8.10) of anemia cases were attributed to having no media exposure and maternal anemia, respectively. Similarly, the proportion of anemia cases in the study population that could be attributed to stunting and underweight were estimated to be 1.31% (PAF = 1.31%, 95% CI: 0.65, 2.61) and 0.84% (PAF = 0.84, 95% CI: 0.33, 1.18), respectively. Furthermore, an estimated 1.19% (PAF = 1.19%, 95% CI: 0.88, 1.50) and 2.26% (PAF = 2.26%, 95% CI: 1.84, 2.69) of cases of anemia among children aged 6–59 months could be attributed to having diarrhea and fever within two weeks preceding the survey, respectively. The proportion of anemia cases among children aged 6–59 months attributed to unimproved toile facility was estimated to be 1.02% (PAF = 1.02%, 95% CI: 0.51, 1.54) (Table 2).

## Discussion

Our study examined the impacts of modifiable risk factors on anemia among children aged 6–59 months in SSA based on DHS data from 27 sub-Saharan African countries. The findings of our study revealed that born to mothers having no formal education, primary education, or secondary education, belonged to the poorest, poorer, and middle household wealth, had no media exposure, unimproved toilet facilities, were stunted, were underweight, born to anemic mothers, having diarrhea and fever within two weeks were significantly associated with higher prevalence odds of anemia among under-five children. These findings were in line with previously published studies [1,51–53].

The prevalence of anemia among children aged 6–59 months in SSA was 61.99% (95% CI: 61.73, 62.27). It was higher than studies conducted in Sri Lanka [54], Nepal [55] and Bangladesh [56]. It could be due to the high prevalence of undernutrition and micronutrient deficiencies such as iron, folate, vitamin B9, and B12, which could cause anemia [57,58]. In addition, sub-Saharan African countries are hotspot areas for anemia-causing infectious diseases such as malaria, tuberculosis, parasitic infections, HIV/AIDS, and others [59].

We estimated the population-attributable fraction of modifiable risk factors to quantify the proportion of anemia that could be prevented if the risk factors were reduced to zero in the population. It measures the overall effect of risk factors on anemia among children aged 6–59 months at the population level. In sub-Saharan Africa, childhood anemia is a major public health problem, with its magnitude varying across countries. The progress toward reducing the prevalence of anemia among under-five children demands multi-dimensional public health interventions that maximize existing resources and capabilities.

Our study highlighted the need to prioritize resources based on the order of importance of modifiable risk factors identified to be significantly associated with anemia in children aged 6–59 months such as maternal anemia (7.37%), lack of formal education for mothers (5.86%), having primary education (3.71%), being in the poorest household (3.52%), having secondary education (2.68%), being in the poorer household (2.53%), having history of fever within two weeks (2.26%), being in the middle household wealth (1.99%), being in the richer household (1.51%), being stunted (1.31%), having history of diarrhea within two weeks (1.19%), lack of improved toilet facility (1.02%), being underweight (0.84%) and lack of media exposure (0.76%). These findings can inform funding, public health practice, and policy priorities to address anemia in children.

Consistent with study findings reported in SSA [4] and Bangladesh [56], mothers with no formal education, and primary and secondary education positively contributed to childhood anemia compared to mothers with higher education. It could be because educated mothers are more likely to understand the value of a balanced diet, children’s nutritional needs, and the sources of essential nutrients such as iron, folate, and vitamins [60]. Furthermore, educated mothers are more likely to seek timely medical care for their children, ensuring early detection and treatment of illnesses that can lead to anemia, as well as ensuring that their children are fully vaccinated, lowering the risk of anemia-causing infections [61]. Belonged to a disadvantaged household significantly contributed to anemia in children aged 6–59 months. It is supported by previous research findings [51,62], that it is possible that children from low-income families lack access to dietary diversity and may suffer from micronutrient deficiencies such as iron, folate, and vitamins [63]. Poverty can lead to food insecurity, poor hygiene, and inadequate child care. This leads to malnutrition, including iron deficiency anemia. In addition, poverty is strongly linked to limited access to health care for childhood illnesses and the inability to purchase nutrient-dense foods [64].

Consistent with previous studies reported in Nepal [65] and Indonesia [66], diarrhea and febrile illness positively contributed to anemia among children aged 6–59 months. It could be because diarrhea causes significant nutrient loss, including folate, iron, and vitamins, and febrile illnesses like malaria and visceral leishmaniasis can cause anemia directly or indirectly [67]. Another important risk factor contributed to anemia in children aged 6–59 months in this study was stunting and underweight. Being stunted and underweight positively contributed to anemia in children aged 6–59 months. It is consistent with research findings from Africa [68] and Indonesia [69], and it could be because undernourished children are more likely to have micronutrient deficiencies such as iron, folate, and vitamin B2, all of which are essential for the production of red blood cells [70]. Furthermore, malnourished children have weakened immune systems and are more vulnerable to infectious diseases such as tuberculosis, hookworm, visceral leishmaniasis, and other chronic diseases, all of which cause anemia [71].

Another important public health risk factor that contributed to anemia in children was maternal anemia. It has a very large contribution to anemia in children aged 6–59 months in SSA. It was supported by previous studies [52,72]. According to available evidence, anemic mothers usually reside in the most disadvantaged households, making it difficult for them to purchase and provide diverse food for their children, which can lead to anemia in children [73]. Furthermore, mothers share a similar socioeconomic environment, including dietary habits and quality of life, as well as exposure to diseases such as malaria and other anemia-causing diseases [74].

### Strengths and limitations

This study has several strengths. Firstly, this study is based on large nationally representative DHS datasets that enhanced the power of the study and external validity of our findings to SSA. Secondly, robust statistical models such as multilevel modified Poisson regression model and PAF to estimate the contribution of modifiable risk factors for anemia. Apart from the strengths, this study has the following limitations. First, this study was based on the DHS data, which is a cross-sectional study therefore we are unable to establish a cause-effect relationship between the risk factors and anemia. Second, important variables such as underlying infectious diseases (malaria, tuberculosis, HIV/AIDS, intestinal parasites) and underlying medical conditions were not considered as DHS did not contain these variables.

## Conclusion

Anemia among children aged 6–59 months in SSA has been identified as a major public health concern. This study focused on the key modifiable risk factors previously associated with anemia in children aged 6–59 months in SSA. The estimated public health impact of each modifiable risk factor on childhood anemia was significant. In SSA, maternal anemia, maternal education, household wealth, child nutritional status, diarrheal, and febrile illness accounted for nearly two-thirds of anemia among children aged 6–59 months. Our study’s findings highlighted the importance of public health interventions aimed at anemic mothers, low-income, poor educational status, undernourished children, and children with common childhood illnesses to significantly reduce the incidence of anemia in children.

## Recommendation

Strengthening maternal health programs to prevent and manage anemia in mothers, improving access to quality education for women, implementing economic empowerment initiatives for low-income households, and enhancing child nutrition programs are essential strategies. For future research, we recommend longitudinal studies to establish causal relationships between these risk factors and childhood anemia, as well as intervention-based studies to evaluate the effectiveness of targeted public health programs.

## Supporting information

S1 File Stata do file for population attributable fraction. (DO)

## Abbreviations

aPOR: Adjusted Prevalence Odds Ratio
CI: Confidence Interval
DHS: Demographic and Health Survey
EAs: Enumeration Areas
ICC: Intra-cluster Correlation Coefficient
LLR: Log Likelihood Ratio
LRT: Likelihood Ratio Test
SSA: Sub-Saharan Africa
WHO: World Health Organization
